# In Silico Identification of Antiviral Peptides as Potential Leads Against Sudan Ebolavirus VP‐40

**DOI:** 10.1155/bmri/2204127

**Published:** 2026-01-26

**Authors:** Boniface Omara, Kenedy Kiyimba, Fatoumata G. Fofana, Oudou Diabaté, Walter Odur, Daudi Jjingo, Jacob Stanley Iramiot, Peace Draleru, Joan Achia, Muhammad Shafiq, Zaheer Ul-Haq, Hedmon Okella, Steven Odongo

**Affiliations:** ^1^ Department of Pathology, Faculty of Health Sciences, Busitema University, Mbale, Uganda, busitema.ac.ug; ^2^ Department of Biotechnical and Diagnostic Sciences, College of Veterinary Medicine, Animal Resources and Biosecurity, Makerere University, Kampala, Uganda, mak.ac.ug; ^3^ Department of Pharmacology and Therapeutics, Faculty of Health Sciences, Busitema University, Mbale, Uganda, busitema.ac.ug; ^4^ Natural Product Research and Innovation Centre, Faculty of Health Sciences, Busitema University, Mbale, Uganda, busitema.ac.ug; ^5^ African Centre of Excellence in Bioinformatics and Data Sciences (ACE-Mali), University of Sciences Techniques and Technologies of Bamako (USTTB), Bamako, Mali; ^6^ African Centre of Excellence in Bioinformatics and Data Intensive Sciences (ACE-Uganda) Infectious Diseases Institute (IDI), McKinnell Knowledge Centre, Makerere University, Kampala, Uganda, mak.ac.ug; ^7^ The Infectious Diseases Institute, Makerere University, Kampala, Uganda, mak.ac.ug; ^8^ Department of Computer Science, College of Computing and Information Sciences, Makerere University, Kampala, Uganda, mak.ac.ug; ^9^ Department of Microbiology and Immunology, Faculty of Health Sciences, Busitema University, Mbale, Uganda, busitema.ac.ug; ^10^ Department of Anatomy, Faculty of Health Sciences, Busitema University, Mbale, Uganda, busitema.ac.ug; ^11^ H. E. J. Research Institute of Chemistry, International Centre of Chemical and Biological Sciences, University of Karachi, Karachi, Pakistan, uok.edu.pk; ^12^ Pharm-Biotechnology and Traditional Medicine Centre, Mbarara University of Science and Technology, Mbarara, Uganda, must.ac.ug

**Keywords:** antiviral peptides, molecular docking, molecular dynamics simulation, Sudan Ebolavirus, therapeutic leads

## Abstract

The continued reemergence of Ebola virus epidemics remains a global health concern, largely due to limited therapeutic interventions. This study is aimed at identifying and characterizing antiviral peptides as potential lead candidates against the Sudan Ebola virus. We retrieved antiviral peptides from the AVPdb and designed novel peptides from them using support vector machine, RF, and discriminant analysis algorithms. The toxicity and allergenicity predictions were performed using ToxinPred, ADMETLab 3.0, Allertop, and AllergenFP web servers, respectively. The 3D structures of selected peptides were modeled using PEP‐FOLD and I‐TASSER and validated using ProSA and PROCHECK web servers. The best peptide models were docked against the Sudan Ebola virus VP‐40 protein using HDOCK and ClusPro. Molecular dynamics (MD) simulations were then carried out in GROMACS 2024.2. Out of 170 designed motifs, 30 exhibited antiviral potential with antiviral scores ranging from 0.506 to 1.000. Among the predicted antiviral peptides, five demonstrated favorable stability, nontoxicity, and nonallergenic properties. PEP‐FOLD produced more stable peptide structures than I‐TASSER, with over 84.6% of their amino acids in the most favorable region. Binding energies ranged from −252.39 to −145.83 kcal/mol (HDOCK) and from −887.7 to −538.7 units (ClusPro). The MD simulations confirmed high stability, with motif A10_M showing the strongest binding and structural compactness. Five peptides show strong potential as therapeutic leads against Sudan Ebola virus; however, further experimental validation is recommended.

## 1. Introduction

The Ebola virus disease is one of the deadliest infectious diseases globally, with a mortality rate of up to 90% and an average case fatality rate (CFR) of approximately 50%, varying from 25%–90% in past outbreaks [1]. Ebola viruses belong to the viral order—Mononegavirales, family Filoviridae, and genus—*Ebolavirus* with six identified species known: Zaire ebolavirus (ZEBOV), Sudan ebolavirus (SEBOV), Bundibugyo ebolavirus (BEBOV), Reston ebolavirus (RESTV), Taï Forest virus (TAFV), and the recently discovered Bombali virus (BOMV) [2], four of which can cause human disease (Bundibugyo, Sudan, Taï Forest, and Zaire viruses) [2]. The name of the family Filoviridae originates from the Latin word “*filum*” or thread; the virion shape resembles a twisted thread when viewed under an electron microscope [3]. The genome of Ebola virus is ~19 kb long, linear, nonsegmented negative‐sense (NNS), ssRNA, encoding seven genes [4]. Each gene contains a single open reading frame (ORF) except the GP gene. In contrast, the GP gene consists of three overlapping ORFs [5]. During the assembly, viral RNA forms a ribonucleoprotein (RNP) complex with NP, L, VP‐30, VP‐35, VP‐40, and VP‐24 [6], which appears as a helical nucleocapsid (NC) [7]. NC protects the viral RNA from degradation by endonucleases and the host′s immune response [8]. The African fruit bats of the family Pteropdidae serve as a wildlife reservoir in nature for ZEBOV and MARV [9–11]. Bats carrying the virus transmit it to monkeys, chimpanzees [12], and humans. Human‐to‐human transmission occurs through contact with a patient′s blood and bodily fluids [13]. Ebola was first identified in 1976 when two simultaneous outbreaks occurred in South Sudan and the Democratic Republic of Congo (DRC) [1].

Ebola outbreaks have most commonly been caused by the ZEBOV and SEBOV [14]. The WHO estimates that Ebola virus has resulted in about 11,500 deaths worldwide [1]. Sudan and Uganda have predominantly been affected by SEBOV outbreaks [4]. Since 2000, Uganda has encountered four outbreaks of the Ebola virus disease caused by SEBOV; the outbreak from October 2000 to January 2001 that occurred in Gulu, Masindi, and Mbarara districts resulted in 425 cases and 224 (53%) deaths [15]. The Kibaale district outbreak from January 2012 to August 2012 resulted in 24 cases and 17 (71%) deaths. On the 20th of September 2022, Uganda′s health officials declared the fifth Ebola disease outbreak caused by SEBOV [16]. The first case was detected in the Mubende district among people living around a gold mine [17]. During this outbreak, as of December 1, 2022, the Uganda Ministry of Health reported 142 confirmed cases (22 Probable) and 55 (39%) deaths [17]. Of these, healthcare workers accounted for (19) 13.3% of the cases and 7 (12.7%) of all deaths, CFR of 36.8% [17], and an estimated number of children orphaned or left unaccompanied [18].

During outbreaks, the number of cases and deaths from the SEBOV continues to rise since there is no approved vaccine or drug to prevent and treat the deadly disease [1]. The interventions for effective management of the Ebola outbreak rely on case management, surveillance, and contact tracing, alongside the provision of optimal laboratory diagnostic services. Equally critical are infection prevention and control measures implemented at both healthcare and community levels. Additional strategies include the conduct of safe and dignified burials, sustained community engagements, and social mobilization efforts. Furthermore, public health measures such as movement restrictions, including lockdowns, curfews, quarantine, halting flights from affected regions, and mandatory testing of travelers from high‐risk areas, among others, [1] have been employed to mitigate transmission and contain outbreaks.

Several therapeutic approaches have been explored for Ebola virus infections, each with distinct advantages and limitations. The FDA has also approved two monoclonal antibodies, InmazedTM (atoltivimab/maftivimab/odesivimab) and EbangaTM (ansuvimab), which demonstrated efficacy against ZEBOV during clinical trials in the 2018–2020 DRC outbreak [19, 20]. Although monoclonal antibodies offer high specificity and have shown significant mortality reduction (from 67% to 35% in some studies), their utility is limited by strain‐specific activity, as neither has demonstrated effectiveness against SEBOV [21]. Furthermore, monoclonal antibodies require cold‐chain storage, which is expensive to manufacture and may trigger immunogenic responses with repeated administration [22].

Small‐molecule antivirals represent another therapeutic approach. Remdesivir is a nucleotide analog that inhibits viral RNA‐dependent RNA polymerase, which showed promise in preclinical studies against ZEBOV but demonstrated limited clinical efficacy in controlled trials [23]. Galidesivir (BCX4430) is an adenosine analog, which has exhibited in vitro antiviral activity against SEBOV and broad‐spectrum activity against multiple RNA viruses [24]. However, small molecules face challenges, including rapid viral mutation leading to drug resistance, potential off‐target effects, and variable pharmacokinetic properties that may limit tissue penetration [25]. In addition, the high mutation rate of RNA viruses can quickly compromise the efficacy of small‐molecule inhibitors targeting specific viral proteins [26].

RNA interference (RNAi) therapeutics, particularly small interfering RNAs (siRNAs), have demonstrated antiviral activity by targeting specific viral mRNA sequences for degradation [27]. TKM‐Ebola (not using anymore) showed protective effects in nonhuman primate models but faced challenges in clinical translation, including delivery efficiency, stability, biological fluids, potential off‐target effects, and the requirement for lipid nanoparticle formulations [28]. Moreover, siRNA therapeutics are highly sequence specific, limiting their effectiveness across different Ebola virus species [29].

Ion channel inhibitors, such as amiodarone and verapamil, have been investigated based on the hypothesis that they can block viral entry by disrupting endosomal acidification [30]. Although these repurposed drugs offer the advantage of established safety profiles and immediate availability, their mechanism is indirect and may lack the potency required for effective monotherapy against SEBOV [31]. Further, their use is complicated by cardiovascular side effects and drug–drug interactions [32].

In contrast, antiviral peptides (AVPs) represent a promising therapeutic modality with several distinct advantages. AVPs can target multiple stages of the viral life cycle, including viral attachment, membrane fusion, entry, and replication [33]. Their mechanism of action often involves direct disruption of viral membranes or interference with protein–protein interactions, making them less susceptible to resistance development compared to small molecules [34]. Peptides can be designed to target conserved viral regions, potentially offering broad‐spectrum activity across different Ebola virus species, including both ZEBOV and SEBOV [35]. Additionally, AVPs offer several other advantages, such as being rationally designed and rapidly synthesized, allowing for quick response to emerging outbreaks [36]. They exhibit low toxicity due to their natural origin or biomimetic design [37]. Also, they can be modified with various chemical moieties to enhance stability, cell penetration, and half‐life [38]. Furthermore, peptides can be produced through chemical synthesis or recombinant expression, potentially reducing manufacturing costs compared to monoclonal antibodies [37]. Interestingly, AVPs face limitations such as susceptibility to proteolytic degradation in biological fluids, potentially limited bioavailability when administered orally, possible immunogenicity with repeated administration, and challenges in large‐scale manufacturing for pandemic response [39].

Despite the challenges related to delivery, stability, and half‐life, recent advances in peptide engineering, such as cyclization, incorporation of nonnatural amino acids, PEGylation, and development of cell‐penetrating peptide conjugates, have shown promise in overcoming many of the limitations [38–42]. Previous studies indicate limited attempts to explore promising potential AVPs, such as antiviral Rift Valley fever virus peptide (US9556237B2) [43] and cholesterol‐conjugated stapled peptides for the management of Ebola viruses [44]. Notably, Galidesivir and *Δ*‐peptide immunoadhesins [45] derived from the Ebola virus sGP have been reported to inhibit the activity of the SEBOV. Whereas these compounds have demonstrated potential against Ebola virus, the limited efficacy of the current treatment against SEBOV highlights the need for alternative therapeutic options. Although previous research has explored peptides against the Ebola virus, our study takes a novel approach by designing peptides from experimentally validated AVPs found in the AVP database, which researchers have not previously studied for the SEBOV strain. In addition, the nature and interaction of such peptides with known SEBOV VP‐40 remain unknown in the quest for novel drug candidates against SEBOV. Thus, this study is aimed at identifying and characterizing novel AVPs for SEBOV using computational approaches to provide treatment options for this reemerging infectious disease.

## 2. Materials and Methods

**Figure 1:**
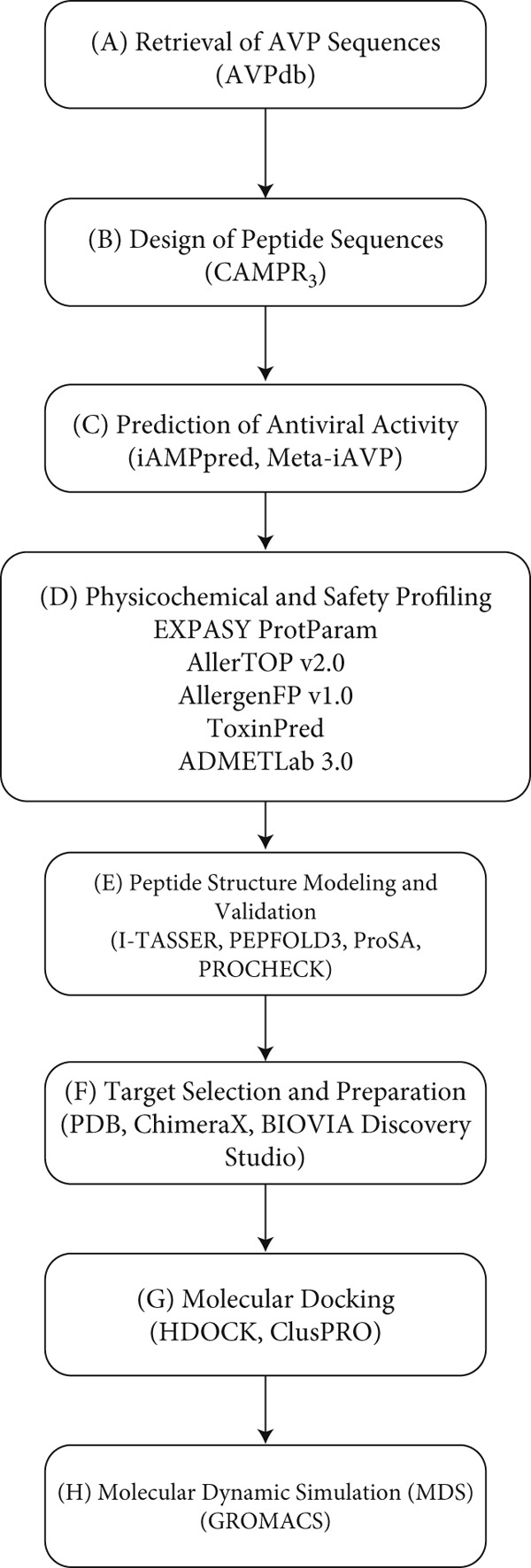
Workflow for the in silico identification of AVPs. (a) Retrieval of AVPs from the AVPdb, (b) design of peptide sequences, (c) prediction of antiviral activity, (d) physicochemical and safety profiling, (e) peptide structure modeling and validation, (f) target selection and preparation, (g) molecular docking, and (h) molecular dynamics simulation.

This current study involves several steps, from the retrieval of AVPs to molecular dynamics (MD) simulations. These steps are shown in Figure 1.

### 2.1. Retrieval of AVP Sequences

All AVPs cataloged in the Antiviral Peptide Database, a comprehensive and well‐curated database for AVPs (available at http://crdd.osdd.net/servers/avpd), were retrieved on October 2nd, 2024 [46]. Thereafter, peptides that were previously found to have antiviral activity against the Ebola virus were selected for motif design. We used an experimentally guided selection approach to identify peptides annotated in the AVPdb and the primary literature as having experimental antiviral activity against the Ebola virus species.

### 2.2. Design of Peptide Sequences

The selected sequences in FASTA file format were submitted to the web server for the Collection of Anti‐Microbial Peptides (CAMPR_3_) for designing and identification of potential antimicrobial peptides [47]. CAMPR_3_ was visited at https://www.camp3.bicnirrh.res.in/index.php. The CAMPR_3_ contains information on conserved sequence signatures captured as patterns and hidden Markov models (HMMs) in 1386 AMPs represented by 45 families with their prediction algorithms incorporated. These algorithms are based on support vector machines (SVMs), random forests (RFs), artificial neural networks (ANNs), and discriminant analysis (DA).

### 2.3. Prediction of the Antiviral Activity of the Designed Peptides

The antiviral activity of the generated motif sequences of a particular peptide was predicted using the *iAMPpred* online server [48]. This server contains a SVM for the prediction of AMPs and non‐AMPs. Further, we confirmed the antiviral activity of the selected peptides using Meta‐iAVP, a sequence‐based meta‐predictor for improving the prediction of AVPs using effective feature representation [49]. The meta‐iAVP webserver employs ensemble machine learning models to predict AVPs based on probability scores. The threshold of 0.5 is recommended in the meta‐iAVP documentation as the default cut‐off for classifying peptides as potential antiviral candidates, since scores ≥ 0.5 indicate that the peptide is more likely to possess antiviral activity [49]. Therefore, only peptides showing antiviral activity ≥ 0.5 were selected for physicochemical and safety profiling, whereas those with no antiviral activity < 0.5 were excluded.

### 2.4. Physicochemical and Safety Profiling

#### 2.4.1. Physicochemical Properties

The physicochemical properties of the peptide sequences, including hydrophobicity, instability, isoelectric point, GRAVY score, net charge, and molecular weights, were checked using the ExPASy ProtParam3 tool (https://web.expasy.org/protparam/) [50].Sequences with an instability index below 40 were classified as stable, whereas those above 40 were considered unstable.

#### 2.4.2. Allergenicity

The allergenicity was checked using AllerTOP.v2.0 [51] at http://www.pharmfac.net/allertop and AllergenFP.v1.0 [52] at http://ddg-pharmfac.net/Allergen FP. AllergenFP.v1.0 uses amino acid descriptors and auto‐covariance and cross‐covariance transformation of protein sequences into uniform equal‐length vectors to predict allergens. Both servers classify sequences as either allergen or nonallergen using description‐based machine learning approaches. We considered proteins not having allergic properties by both prediction methods for further analysis.

#### 2.4.3. Toxicity

We predicted the toxicity using the ToxinPred server. This server is incorporated with an algorithm that uses motif information for the detection of toxic peptides [53]. This server was visited at http://webs.iiitd.edu.in/raghava/toxinpred/submitfreq_S.php?ran=59761 (accessed on May 7th, 2025). During the development of this software, motifs in toxic peptides were searched by MEME software, and then query sequences were hit with the toxic peptide motif list by MAST software. Specifically, we retained peptides predicted as nontoxic by ToxinPred (SVM < 0). To cross‐validate the toxicity of the peptide, raw sequence files were converted into Simplified Molecular Input Line Entry System (SMILES) structural format using a web‐based tool, PepSMI at https://www.novoprolabs.com/tools/convert-peptide-to-smiles-string (accessed on May 7th, 2025). Later, the ADMETLab 3.0 platform at https://admetlab3.scbdd.com/server/screening (accessed on May 7th, 2025) was used [54]. The platform predicts the toxicity of the peptide based on the corresponding basic information and experimental values of the respective entries at the platform. We only considered peptides consistently classified as nontoxic and nonallergenic across at least two independent tools for downstream analysis.

### 2.5. Peptide Structure Design and Validation

The three‐dimensional structures of putative peptides were predicted using the Iterative Threading Assembly Refinement (I‐TASSER) server at https://zhanglab.ccmb.med.umich.edu/I-TASSER/ [55]. Then, the peptides were modeled using protein templates identified by the Local Meta‐Threading Server (LOMETS) from the PDB library. LOMETS uses multiple threading approaches to align the query protein amino acid sequence against the PDB. We used template proteins with the highest sequence identity and lowest z‐score in the modeling exercise and identified the best models based on their c‐scores. This score is calculated based on the significance of threading template alignments and the convergence parameters of the structure assembly simulations. It ranges from −5 to 2, where a lower score value shows a highly confident model, whereas a higher score shows the reverse. We cross‐validated the peptide 3D structure prediction using an efficient, accurate, and specific web‐based de novo peptide structure prediction tool, PEP‐FOLD v3.5 at https://bioserv.rpbs.univ-paris-diderot.fr/services/PEP-FOLD3/ [56]. Due to limited modeling templates, this tool uses a de novo approach, combining simulated annealing and coarse‐grained force fields to efficiently sample conformational space and predict peptide structures. The evaluation of these peptide structures was carried out in two phases. First, we visited the Protein Structure Analysis (ProSA) web server at https://prosa.services.came.sbg.ac.at/prosa.php [57] to predict the query protein z‐score, local model quality, and residue energy. The z‐score shows the model quality by comparing the query protein z‐score against the z‐score of experimentally validated proteins available in the protein data bank (PDB). In the second phase, PROCHECK was used to measure the stereochemical properties of the modeled peptide motifs [58]. This tool checks the model′s stereochemistry to ensure that it adheres to the expected geometric standards and generates Ramachandran plots, which visualize the allowed regions of dihedral angles in a protein structure.

### 2.6. Target Selection and Preparation

Matrix protein (VP‐40) plays a significant role in the virus lifecycle and was thus selected as a promising therapeutic target. The protein plays a crucial role in regulating the replication, transcription, assembly, and release of viral particles in the host cell, showing high expression [59]. In addition, VP‐40 is highly conserved across different Ebola virus strains, increasing the potential for broad‐spectrum antiviral activity. Inhibiting VP‐40 could effectively disrupt the viral life cycle, reducing the viral load and severity.

The 3D structure of the matrix protein VP‐40 was retrieved from the PDB at https://www.rcsb.org/ [60]. The retrieved target protein was then prepared using BIOVIA Discovery Studio 2021 client v21.1. Here, water molecules and all heteroatoms were removed, missing atoms were replaced, and hydrogen atoms were added.

### 2.7. Molecular Docking

The docking was carried out on the top five potential peptides against VP‐40 using HDOCK [61–65] and ClusPro [66–70] web servers. To enhance the reliability of docking predictions, we adopted a consensus approach and docked peptides using both HDOCK (primary) and ClusPro (validation). Specifically, we centered the docking grid on the active site residues of the VP‐40 receptor, with dimensions set to *x* = 16.425875, *y* = 31.95817, and *z* = 10.624702. These parameters ensured that the binding pocket was adequately covered, allowing the peptides to explore all potential interaction sites within the defined region. The HDOCK server is a highly integrated suite of homology search, template‐based modeling, structure prediction, macromolecular docking, biological information incorporation, and job management for robust and fast protein–protein docking. On the other hand, ClusPro performs a global docking procedure in four folds: motif‐based prediction based on peptide conformation, rigid body docking, scoring based on structural clustering, and final structure minimization. Briefly, the 3D structures of both the receptor protein (retrieved from PDB) and the modeled 3D peptide structures were used as input files for docking. The interactions between the ligands and the VP‐40 target at different docking poses were then unveiled using BIOVIA Discovery Studio 2021 client. The peptide–protein interaction patterns were further evaluated through two‐dimensional (2D) contact map analysis generated by the PDBsum Generate server at (https://www.ebi.ac.uk/thornton-srv/databases/pdbsum/Generate.html) [71] visited on September 26th, 2025. Here, top‐ranked docking models were used as input files.

### 2.8. MD Simulations

MD simulations were performed on the docked protein–peptide complexes using the GROMACS 2024.2 package to investigate the dynamic interactions between the peptides and the target protein [72]. Before proceeding to the system preparation protocols, the missing segments in the protein structure were resolved through the modeler package (version 10.5) [73]. While preparing the protein systems, the GROMOS96 54a7 force field [74] was employed to topologically parameterize the systems because it has been extensively validated and widely employed for biomolecular systems, including protein–peptide complexes. A cubic box was used to contain the protein–peptide complexes, with a 10 Å distance from the box edges [75]. The prepared systems were solvated using the SPC water model, and the genion module of GROMACS was used to incorporate Na^+^ and Cl^−^ counter‐ions for charge neutralization of the solvated systems [76]. Subsequently, the solvated systems were relaxed by minimizing the overall energy with the steepest descent approach, with a maximum of 50,000 steps to remove any unfavorable contacts and solve geometric constraints, and a final force threshold set below 1000 kJ mol^−1^ nm^−1^. After energy minimization, equilibration of the systems was brought about by 5000 ps NVT and an equal run of NPT ensembles to adjust the temperature to 300 K and the pressure to 1 bar, respectively. Lastly, a 200 ns production MD was run for all the systems, and their trajectory data were collected for post‐MD analysis.

## 3. Results

### 3.1. Sequence Retrieval

A total of 2059 peptide sequences were retrieved from the AVP Database (AVPdb), from which 11 peptides were identified as experimentally validated anti‐Ebola peptides. The average amino acid length for the qualified peptide sequences was 32.4, with the longest having 48 amino acids and the shortest having 17 amino acids. Up to 81.81% of these qualified peptides were of Ebola delta origin (Table S1).

### 3.2. AVP Design

Up to 170 peptides were designed from the 11 experimentally validated anti‐Ebola peptide sequences. The peptide sequences generated a varying number of peptides with A3 (Fc‐tagged delta peptide) from the EboV delta peptide, yielding the highest number of peptides (31), whereas A11 from the EboV delta peptide produced the least number of peptides (1) in different positions (Table S2).

### 3.3. Antiviral Activity Predictions

Out of the 170 peptides, 30 exhibited antiviral properties (antiviral probability ≥ 0.5), whereas the rest had an antiviral probability < 0.5. Motifs A5_M and A6_R demonstrated the highest antiviral probability of 1.00, whereas A5_E and A6_F demonstrated the lowest antiviral probability of 0.506 (Table 1).

**Table 1:** Antiviral activity of the generated peptide sequences.

**Sequence Id**	**Position**	**Peptide sequence**	**AVp**
A1_C	3–20	ESLTDRELLLLIARKTCG	0.694
A5_D	5–22	EESPTGPPGSIRTWFQRI	0.906
A5_E	12–19	PGSIRTWFQRIPLGWFHC	0.506
A5_I	16–33	RTWFQRIPLGWFHCTYQK	0.736
A5_K	14–31	SIRTWFQRIPLGWFHCTY	0.998
A5_M	15–32	IRTWFQRIPLGWFHCTYQ	1.000
A5_N	7–24	SPTGPPGSIRTWFQRIPL	0.696
A6_D	5–22	EESPTGPPGSIRTWFQRI	0.906
A6_E	21–38	RIPLGWFHCTYQKGKQHC	0.980
A6_F	12–29	PGSIRTWFQRIPLGWFHC	0.506
A6_I	19–36	FQRIPLGWFHCTYQKGKQ	0.770
A6_L	22–39	IPLGWFHCTYQKGKQHCR	0.980
A6_M	16–33	RTWFQRIPLGWFHCTYQK	0.736
A6_N	20–37	QRIPLGWFHCTYQKGKQH	0.988
A6_P	14–31	SIRTWFQRIPLGWFHCTY	0.998
A6_R	15–32	IRTWFQRIPLGWFHCTYQ	1.000
A6_S	17–34	TWFQRIPLGWFHCTYQKG	0.854
A6_T	7–24	SPTGPPGSIRTWFQRIPL	0.696
A10_B	2–19	FQRIPLGWFHCTYQKGKQ	0.770
A10_C	3–20	QRIPLGWFHCTYQKGKQH	0.988
A10_D	4–21	RIPLGWFHCTYQKGKQHC	0.980
A10_E	5–22	IPLGWFHCTYQKGKQHCR	0.980
A10_F	6–23	PLGWFHCTYQKGKQHCRL	0.964
A10_G	7–24	LGWFHCTYQKGKQHCRLR	0.586
A10_H	8–25	GWFHCTYQKGKQHCRLRI	0.946
A10_I	9–26	WFHCTYQKGKQHCRLRIR	0.932
A10_J	10–27	FHCTYQKGKQHCRLRIRQ	0.544
A10_K	11–28	HCTYQKGKQHCRLRIRQK	0.640
A10_M	13–30	TYQKGKQHCRLRIRQKVE	0.736
A10_N	14–31	YQKGKQHCRLRIRQKVEE	0.588

Abbreviation: AVp, antiviral probability and Sequence Id, sequence identity.

### 3.4. Physicochemical and Safety Profiling

From the 30 promising peptides, 11 were predicted to be nontoxic and without allergen traits, and negative hydrophobicity values ranging from −0.06 to −0.57, a net charge between +0 and +5.50, a molecular weight between 2010.3 and 2352.74, and a pI from 6.50 to 10.90. Also, 1 sequence, A1_C, exhibited a positive gravy of 0.10, whereas the rest exhibited a negative gravy. Further, 10 peptides were predicted as stable, whereas peptide A10_N was predicted to be unstable (Ii > 40). Despite the predicted high instability index (47.96), peptide A10_N was selected for further evaluation because of its promising antiviral probability, nontoxicity, and nonallergenic properties (Table 2).

**Table 2:** Physicochemical properties and safety profiles of computationally predicted antiviral peptides.

**Sequence Id**	**Sequence**	**Charge**	**H**	**G**	**Mw (Da)**	**pI**	**Ii**	**Toxicity**	**Aller**
A1_C	ESLTDRELLLLIARKTCG	0.00	−0.19	0.10	2031.42	6.50	15.96	Nontoxin	PNA
A5_K	SIRTWFQRIPLGWFHCTY	2.50	−0.06	−0.12	2311.96	9.50	24.46	Nontoxin	PNA
A5_M	IRTWFQRIPLGWFHCTYQ	2.50	−0.09	−0.27	2352.74	9.50	24.46	Nontoxin	PNA
A6_I	FQRIPLGWFHCTYQKGKQ	3.50	−0.19	−0.83	2237.61	9.79	36.53	Nontoxin	PNA
A6_N	QRIPLGWFHCTYQKGKQH	4.00	−0.25	−1.16	2227.57	9.79	36.53	Nontoxin	PNA
A6_P	SIRTWFQRIPLGWFHCTY	2.50	−0.06	−0.12	2311.69	9.50	24.46	Nontoxin	PNA
A6_R	IRTWFQRIPLGWFHCTYQ	2.50	−0.09	−0.27	2352.74	9.50	24.46	Nontoxin	PNA
A6_S	TWFQRIPLGWFHCTYQKG	2.50	−0.08	−0.51	2268.62	9.30	19.74	Nontoxin	PNA
A10_C	QRIPLGWFHCTYQKGKQH	4.40	−0.25	−1.16	2227.60	10.20	36.53	Nontoxin	PNA
A10_M	TYQKGKQHCRLRIRQKVE	5.50	−0.55	−1.66	2271.70	10.90	29.85	Nontoxin	PNA
A10_N	YQKGKQHCRLRIRQKVEE	4.50	−0.57	−1.81	2299.71	10.50	47.96	Nontoxin	PNA

Abbreviations: Aller, allergenicity; Da, Dalton; G, GRAVY; H, hydrophobicity; Ii, instability index; Mw, molecular weight; pI, isoelectric point; PNA, probable nonallergens; and Sequence Id, sequence identity.

#### 3.4.1. Toxicity Cross‐Validation

Upon cross‐validation, four peptides, A1_C, A5_K, A10_C, and A10_N, were found to be nonhuman ether a‐go gene blockers (hERG < 0.5), whereas one peptide, A10_M, was found to be a blocker (hERG > 0.5). Further, peptides A5_K, A10_C, and A10_N were found to be nontoxic to the liver, AMES test negative, noncarcinogenic, nonhepatogenic, nonhematogenic, and noncytotoxic, whereas A1_C exhibited toxicity to the liver (DILI > 0.5). Overall, the five peptides (A1_C, A5_K, A10_C, A10_M, and A10_N) exhibited excellent potential (Table 3).

**Table 3:** Toxicity prediction analysis of selected AVPs.

**Property (unit)**	**A1_C**	**A5_K**	**A10_C**	**A10_M**	**A10_N**	**Inference**
hERG blockers	0.000	0.067	0.070	0.512	0.442	> 0.5: Blocker; < 0.5: Nonblocker
DILI	0.674	0.130	0.001	0.003	0.000	> 0.5: Toxic to the liver; < 0.5 nontoxic to the liver
AMES toxicity test	0.008	0.010	0.005	0.010	0.003	> 0.5: Positive; < 0.5: Negative
Carcinogenicity	0.000	0.000	0.000	0.000	0.000	> 0.5: Carcinogen; < 0.5: Noncarcinogen
Rat oral acute toxicity	0.000	0.000	0.005	0.011	0.005	> 0.5: Toxic; < 0.5 Non‐toxic
Human hepatoxicity	0.069	0.214	0.233	0.148	0.114	> 0.5: Hepatotoxic; < 0.5: Nonhepatotoxic
Hematotoxicity	0.005	0.000	0.002	0.000	0.000	> 0.5: Hematotoxic; < 0.5: Nonhematotoxic
Hek cytotoxicity	0.000	0.001	0.001	0.003	0.001	> 0.5: Cytotoxic; < 0.5: Noncytotoxic

Abbreviations: AMES, AMES test; DILI, drug‐induced liver injury, Hek, human embryonic kidney; hERG, human Ether‐a‐go‐go‐Related Gene; Hek, Human Embryonic Kidney.

### 3.5. Peptide Structure Design and Validation

Here, PEP‐FOLD predictions produced 10 models for peptides A1_C, A5_K, A10_C, A10_M, and A10_N. Model_1 for each of the peptides was recognized as the best model, with their released coarse‐grained optimized potential for efficient structure prediction energy and Apollo melting temperature (tm) scores ranging from −33.33 to −15.19 (sOPEP) and 0.792–0.256 (tm), respectively. In contrast, the I‐TASSER predictions produced five models for A1_C, A10_C, A5_K, 3 models for A10_M, and one model for A10_N peptides and had c‐scores ranging between −1.47 and −0.01 (Table S3). When evaluated for general structure quality, the Ramachandran plot demonstrated that the PEP‐FOLD modeled structures for A1_C, A10_M, and A10_N had all their residues (100%) in the most favored region, whereas A10_C and A5_K had 84.6% and 85.7% in the most favored region and 7.7% and 14.3% in the additionally allowed region and the disallowed region, respectively, with none in the generally allowed region (Figures 2 and 3, respectively). Upon subjecting the I‐TASSER and PEP‐FOLD best models to structure quality evaluations, PEP‐FOLD models showed a better quality than the I‐TASSER models (Table 4). In addition, cross‐validation with ProSA showed that all model z‐scores were in the same range as the z‐score of experimentally validated proteins and were thus considered accurate. Given that ProSA yielded analogous z‐scores, we massively relied on Ramachandran plots. Models with the most residues in the most favored region, ≥ 84.6%, were qualified as the best models and were selected for docking and MD simulation exercises.

Figure 2Peptide A10_C PEP‐FOLD predicted 3D structure homology models, Ramachandran, and ProSA validation plots. (a) Stick format of modeled A10_C, (b) Ramachandran plot for the A10_C peptide, and (c) peptide A10_C ProSA z‐score. The PEP‐FOLD‐modeled peptide A10_C had 84.6% residues in the favored region, 7.7% in both the additionally allowed regions and the disallowed region, and 0.0% in the generally allowed region of the Ramachandran plot with a z‐score value of −1.21. Red regions (A, B, and L) represent locations of residues in the most favored region; yellow regions (a, b, and l) represent locations of residues in additional allowed regions; Citrine regions (~a, ~b, ~l, and ~p) represent locations of residues in generously allowed regions; white regions represent locations of residues in disallowed regions. Most of the amino acid residues in the PEP‐FOLD‐modeled A10_C peptide were in the most favored region (84.6%), with no residue in the disallowed region.

**Figure:**
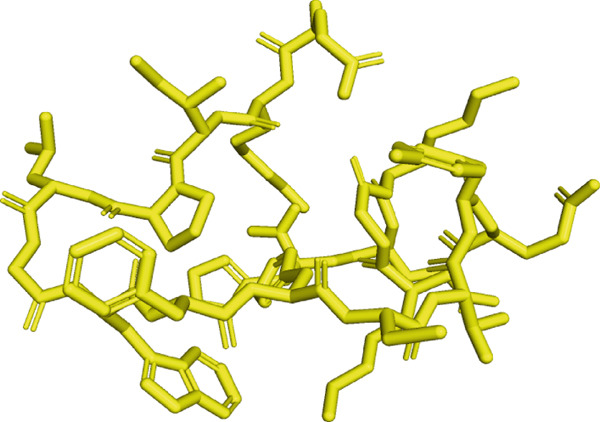
(a)

**Figure:**
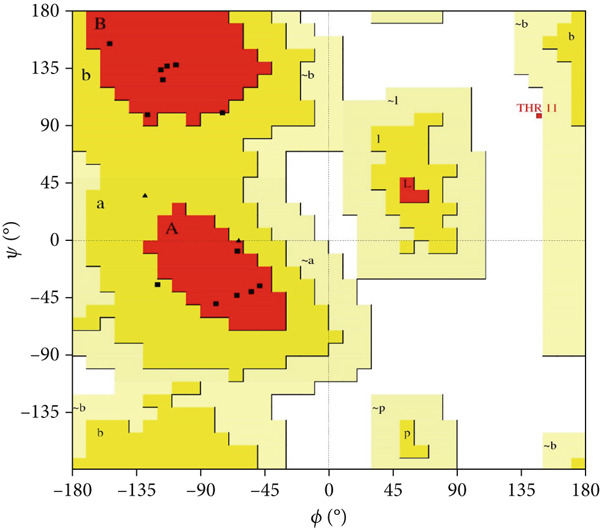
(b)

**Figure:**
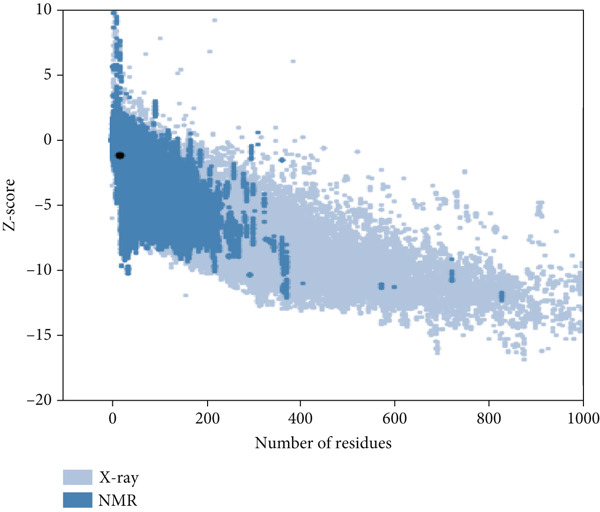
(c)

Figure 3Peptide A5_K PEP‐FOLD predicted peptide 3D structure homology models, Ramachandran, and ProSA validation plots. (a) Stick format of modeled A5_K, (b) Ramachandran plot for the A5_K peptide, and (c) peptide A5_K ProSA z‐score. Peptide‐modeled A5_K had 85.7% residues in the most favored regions, 14.3% in the additionally allowed regions, and 0.0% in both the generally allowed region and the disallowed region of the Ramachandran plot, with a z‐score value of −1.07. Red regions (A, B, and L) represent locations of residues in the most favored region; yellow regions (a, b, and l) represent locations of residues in additional allowed regions; citrine regions (~a, ~b, ~l, and ~p) represent locations of residues in generously allowed regions; and white regions represent locations of residues in disallowed regions. Most of the amino acid residues in the PEP‐FOLD‐modeled A5_K peptide were in the most favored region (85.7%), with no residue in the disallowed region.

**Figure:**
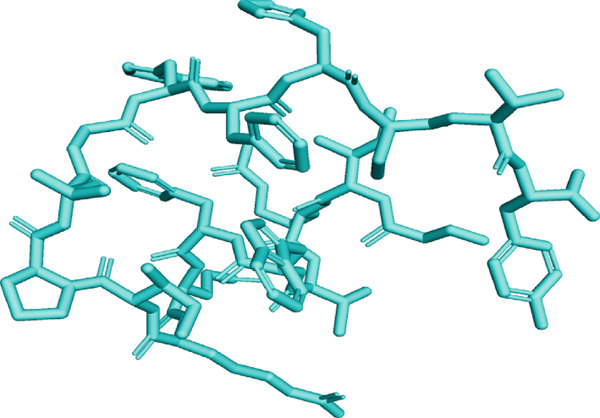
(a)

**Figure:**
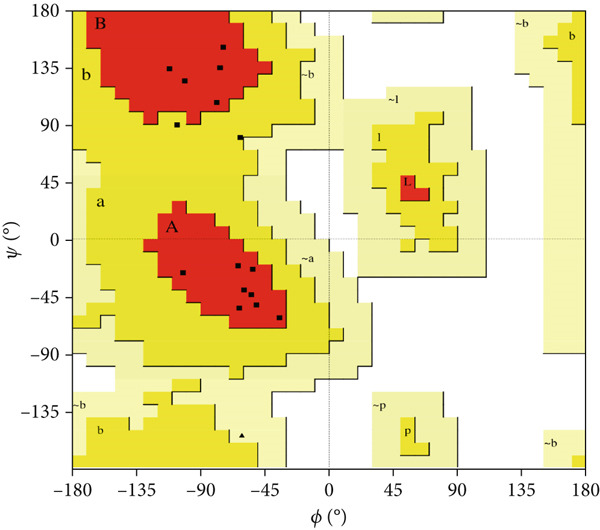
(b)

**Figure:**
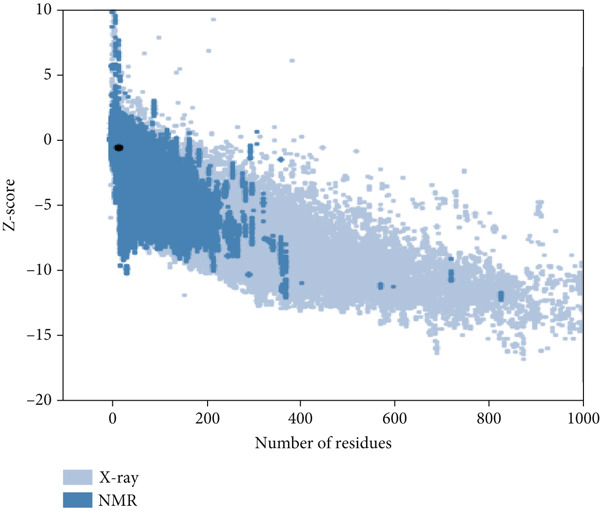
(c)

**Table 4:** Evaluation scores of selected antiviral peptides′ 3D structures predicted.

**Tool**	**Parameters**	**Models**	**A1_C**	**A5_K**	**A10_C**	**A10_M**	**A10_N**
Ramachandran Plot	Residues in most favored regions (%)	I‐TASSER	75.00	42.29	61.50	100	93.30
		PEP‐FOLD	100	85.70	84.60	100	100
	Residues in the additional allowed regions (%)	I‐TASSER	18.80	57.10	30.80	0.00	6.70
		PEP‐FOLD	0.00	14.30	7.70	0.00	0.00
	Residues in the generally allowed regions (%)	I‐TASSER	6.20	0.00	7.70	0.00	0.00
		PEP‐FOLD	0.00	0.00	0.00	0.00	0.00
	Residues in the disallowed regions (%)	I‐TASSER	0.00	0.00	0.00	0.00	0.00
		PEP‐FOLD	0.00	0.00	7.70	0.00	0.00

**ProSA**	Z‐score	I‐TASSER	−1.63	−0.12	−0.49	−0.79	−0.01
		PEP‐FOLD	−1.82	−0.65	−1.21	−1.82	−1.33

Abbreviations: ProSA, Protein Structure Analysis and 3D, Three‐Dimensional.

### 3.6. Target Selection and Preparation

We retrieved the 3‐dimensional structure of the SEBOV protein receptor VP‐40 (PDB ID: 3tcq) from the PDB. Water molecules and all heteroatoms were removed (Figure S1).

### 3.7. Molecular Docking

Docking exercise with HDOCK revealed that peptides were able to bind with low docking energies (ranging from −252.39 to −145.83 kcal/mol), showing their high affinity with the selected target protein. The affinity of peptide motif A5_K was the highest at −252.39 kcal/mol, whereas that of A1_C was the lowest at −145.83 kcal/mol within chains of the target protein (PDB ID: 3tcq). Upon cross‐validation of the docking results using the ClusPro web server, the ClusPro coefficient weight scores ranged from −538.7 to −887.7 units. The coefficient weight score of peptide A10_C was the highest at −887.7 units, whereas A10_M was the smallest at −538.7 units (Table 5).

**Table 5:** Docking energies and scores of the top ligands against the target protein using HDOCK and ClusPro.

**Peptide Id**	**Energies with HDOCK (kcal/Mol)**	**Coefficient weight score with ClusPro (A.U)**
A10_C	−214.91	−887.7
A5_K	−252.39	−860.4
A10_N	−183.33	−666.3
A10_M	−190.95	−538.7
A1_C	−145.83	−635.5

Abbreviations: A.U, arbitrary energy units; kcal/mol, kilocalories per mole; Peptide Id, peptide identity.

#### 3.7.1. Ligand–Protein Molecular Interactions

The peptide A10_C formed three hydrogen bonds with Pro 97 (1), Asp193 (1), and Lys 212 (1), and extensive hydrophobic contacts, indicating a compact and stable binding interface **(**Figure 4a). Similarly, the A10_N formed three hydrogen bonds with Ala156 (1), Leu218 (1), and Pro286 (1), supported by hydrophobic interactions, suggesting a moderately stable complex formation (Figure 4b).

Figure 4LigPlot+ analysis of protein–ligand molecular interactions. (a) 2D diagram of A10_C molecular interaction and (b) 2D diagram of A10_N molecular interaction. The green dashed lines represent the hydrogen bonds between the ligand and the protein; the pink semicircular pokes represent the nonligand residues involved in the hydrophobic contacts.

**Figure:**
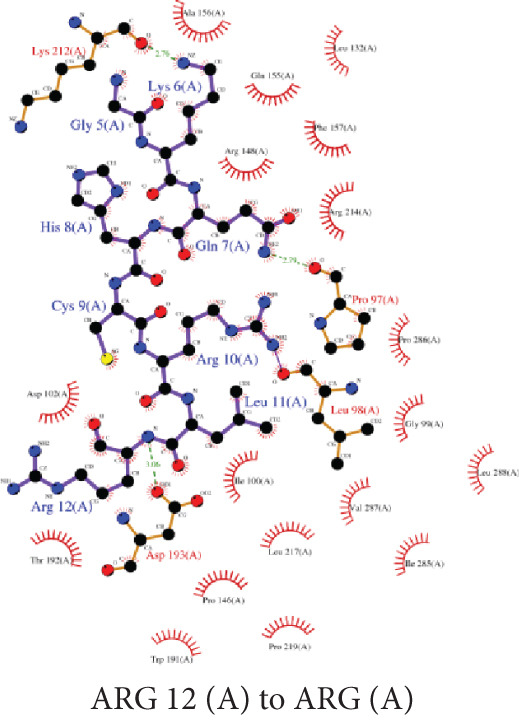
(a)

**Figure:**
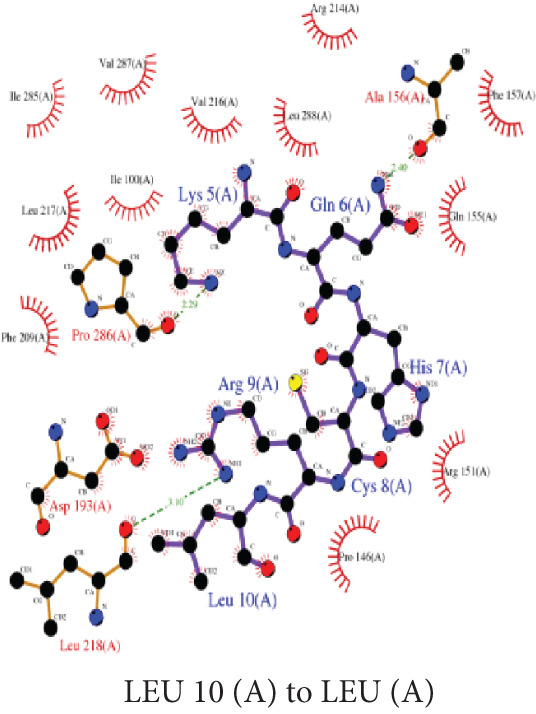
(b)

### 3.8. MD Simulations

For a better understanding of the effect of ligand binding on the target VP‐40 protein, we studied the protein–peptide complexes obtained by molecular docking for their dynamic analysis by MD simulation. The trajectories obtained by MD simulations were monitored to analyze the system stability by recording the root mean square deviation (RMSD). Additionally, the root mean square fluctuations (RMSFs) of the protein residues were analyzed to assess the dynamic flexibility of the protein in each system, and the compactness of the protein structure as a result of the peptide binding was assessed through the radius of gyration (RoG) [76].

**Figure 5:**
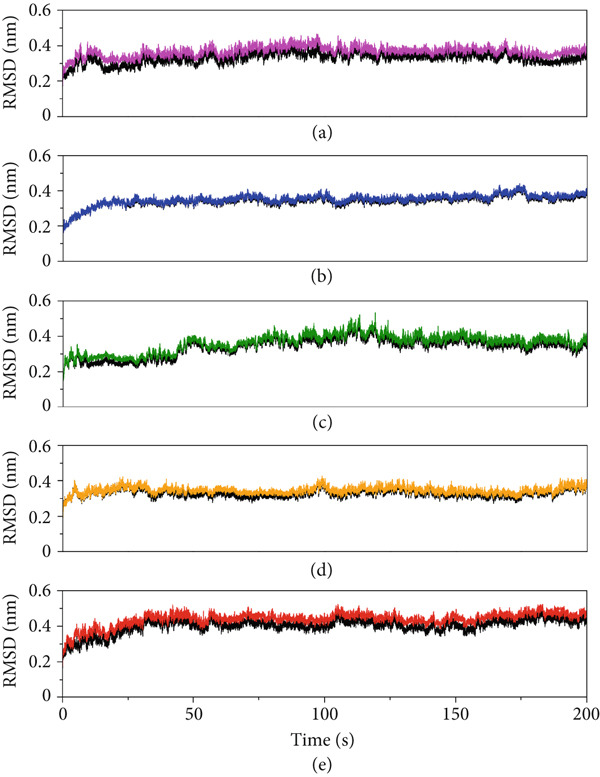
Root mean square deviation (RMSD) plots of the protein backbone in peptide–protein (colored graphs) and apo protein (black graphs) structures. (a) A10_C, (b) A10_M, (c) A10_N, (d) A1_C, and (e) A5_K.

The dynamic stability of the peptide–protein complexes was evaluated using RMSD analyses (Figure 5). Across the 200 ns simulation period, the peptide‐bound VP‐40 systems showed overall stable backbone conformations compared with the apo protein. Notably, the complexes of A10_C and A10_M exhibited relatively lower RMSD fluctuations, suggesting enhanced conformational stability upon peptide binding. The reduced structural deviations observed in these complexes imply that the peptide association contributes to conformational restraint of the VP‐40 protein, a desirable feature for potential inhibitory activity. By contrast, the apo protein displayed greater fluctuations, highlighting the stabilizing influence of the peptides on the protein backbone.

The RMSD of proteins provides a visualization of the dynamic stability in the protein structure with respect to the initial state of the protein. The RMSD plots of the protein backbone in all the studied systems during the 200 ns simulation period (Figure 5). All systems initially displayed a minor increase in RMSD, indicating the equilibration and structural adjustment from their initial conformations, followed by a stable RMSD behavior (between a narrow range of average values between 0.34 and 0.43 nm) in all the systems. Notably, A10_C, A10_M, and A1_C showed a stable behavior with lower values of average (0.35, 0.34, and 0.34 nm, respectively) and maximum (0.46, 0.43, and 0.42 nm, respectively) RMSD, reflecting continuous conformational stability and minimal fluctuations. In contrast, A10_N and A5_K showed slightly higher RMSD (average values 0.36 and 0.43 nm, and maximum values 0.53 and 0.52 nm, respectively), suggesting comparatively higher structural deviations, presumably due to weaker peptide–protein interactions. Overall, a low and stable RMSD trend across all the protein systems implies structural stability of protein–peptide complexes throughout the simulations.

**Figure 6:**
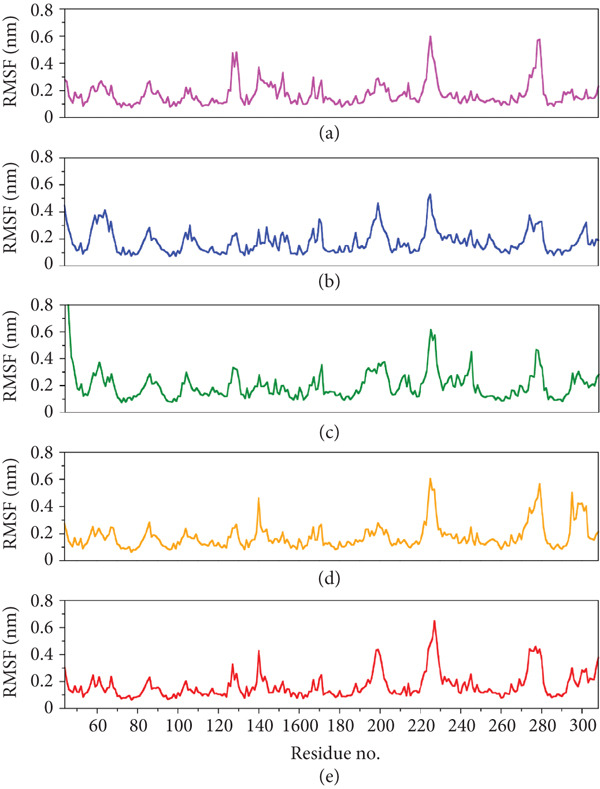
Root mean square fluctuation (RMSF) plots of the protein residues in peptide‐bound protein structures (a) A10_C, (b) A10_M, (c) A10_N, (d) A1_C, and (e) A5_K.

To further analyze the dynamics of the peptide‐bound protein systems, their per‐residue fluctuations were monitored throughout the simulation trajectories using RMSF analysis (Figure 6). Most of the protein residues in all the systems exhibited minor fluctuations (below 0.2 nm), suggesting stability across all the systems. However, loop regions and termini displayed relatively higher RMSF values, which is expected due to their intrinsic flexibility. The protein–peptide complexes of A10_C and A10_N showed the lowest fluctuations in the binding pocket residues, which represent a strong peptide binding in the targeted site. Interestingly, protein in the A1_C complex showed pronounced fluctuations around the residue indexed as 140, potentially implicating conformational rearrangements in response to peptide interaction. A5_K exhibited higher fluctuations in the loop region but retained binding site stability. These differences in RMSF profiles imply that peptide binding affects local flexibility and could influence the biological activity of the complex.

**Figure 7:**
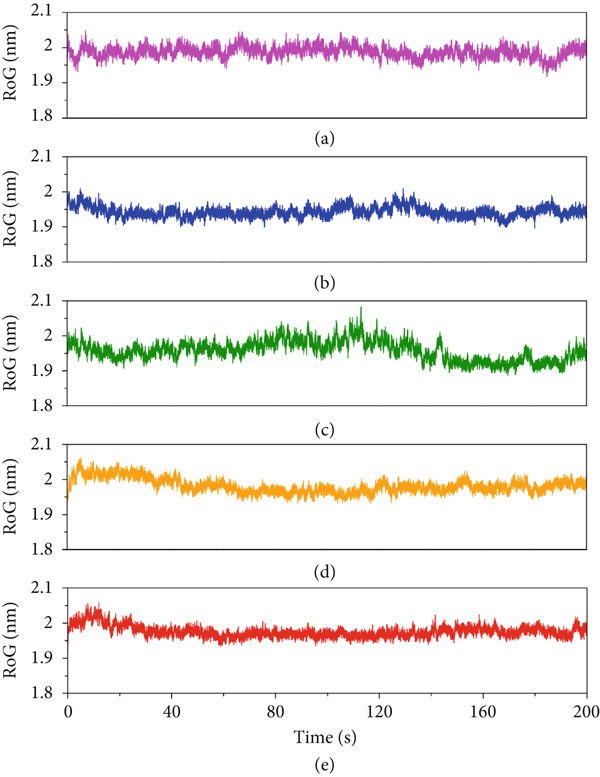
Radius of gyration (RoG) plots of the protein structure in peptide–protein complexes (a) A10_C, (b) A10_M, (c) A10_N, (d) A1_C, and (e) A5_K.

Furthermore, we monitored the folding state of the protein structures using RoG to analyze the compactness of the proteins in the systems over the simulation timeframe (Figure 7). All systems maintained stable RoG profiles throughout the simulations, indicating well‐folded compact protein structures. Interestingly, the protein in the A10_N system demonstrated a compact structure with the lowest RoG values at the start and end of the simulation, suggesting a stable and tightly packed conformation. The consistent RoG trends in the protein structure correlate with stability behaviors observed in RMSD and RMSF plots. Altogether, it can be concluded that all the peptides, particularly A10_C and A10_N, form stable and compact peptide–protein complexes.

**Figure 8:**
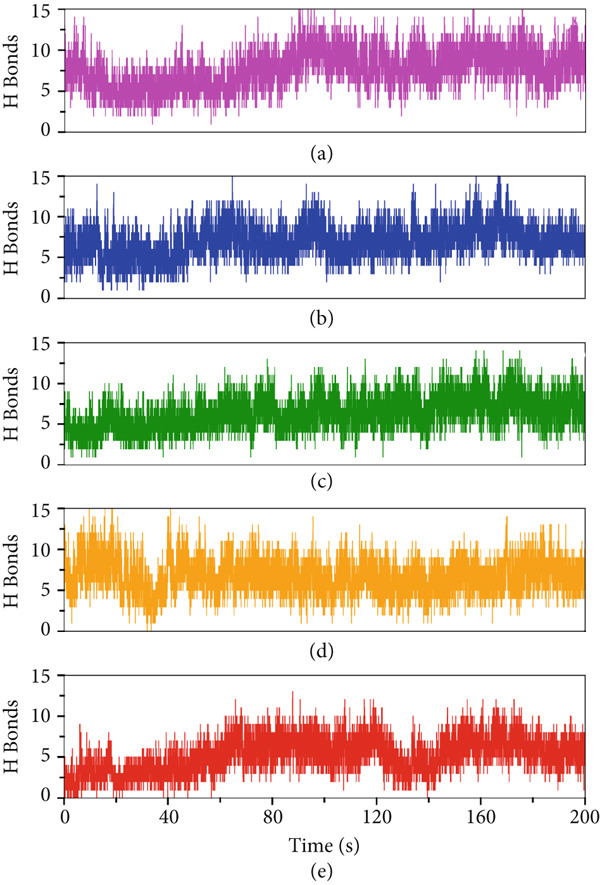
Hydrogen bonding plots of the in peptide–protein systems (a) A10_C, (b) A10_M, (c) A10_N, (d) A1_C, and (e) A5_K.

To further probe the molecular determinants of this stability, we examined hydrogen bond formation within the peptide–protein systems (Figure 8). The plots demonstrate that stable complexes, particularly A10_C and A10_M, maintained a higher and more consistent number of hydrogen bonds throughout the trajectory. These interactions are critical in anchoring the peptides within the VP‐40 binding pocket, supporting both structural stability and specificity of binding. A10_N and A1_C also exhibited moderate hydrogen bond occupancy, though with greater variability, which may explain their slightly higher RMSD values. Interestingly, A5_K formed fewer persistent hydrogen bonds, correlating with its relatively less stable trajectory. In conclusion, these results suggest that peptides with higher hydrogen bond occupancy are more likely to achieve sustained inhibitory interactions with VP‐40.

## 4. Discussion

In the present study, we designed five promising anti‐Ebola peptide leads from already‐known AVPs. One of the key strengths of this study is the fact that we employed all the AVPs ever submitted to the Antiviral Peptide Database. Also, we brought forth the encrypted motifs in such peptides using a combination of machine algorithms, including support vector machine, RF, and discriminant analysis, all housed at the CAMPR_3_ web server [77]. Then, we employed physicochemical parameters at all stages of motif screening. Such efforts enhance the activity of the putative peptides, thus aiding the potential drug candidate search. Although we retrieved all 2059 AVPs in the AVP database, we included only 11 of them (0.53%) in the design. This is because this study focused only on “experimentally validated” AVPs, and only 11 anti‐Ebola peptides are present in the database, which may introduce a bias towards well‐studied peptides. Furthermore, the experimental application of our study′s results could be limited because we used computational models that may not completely represent the complexity of the interactions, and the predicted interactions may not accurately reflect how binding actually works.

The flexibility in the design of motifs yielded up to 30 peptides, 11 of which were nontoxic and without allergen traits, with five having the highest predicted antiviral properties. Following validation using the Meta‐iAVP web server, two Ebola delta peptides were identified with high predicted antiviral probabilities, A5_K (0.998) and A10_C (0.988). This finding demonstrates the ability of AVPs to block EBOV attachment to the host cell, interfere with viral RNA synthesis, and disrupt the interaction between EBOV and the host cell. Our findings agree with several method‐based and AVP discovery studies where high model probabilities reliably enrich for experimentally active sequences [49]. Crucially, several studies targeting filoviruses report that peptide‐based inhibitors can block Ebola virus entry; Fc‐tagged *Δ*‐peptide inhibited GP_1,2_‐mediated entry [45], engineered LL‐37 derivatives impaired EBOV pseudovirion infection [78], and cholesterol‐conjugated stapled fusion inhibitors protected mice from lethal EBOV challenge [44]. Darko et al. [79] demonstrated in their study a low antiviral probability of 0.753 against Ebola virus protein using prediction of activity spectra for substance (PASS) software compared with those reported in our study. In this study, peptides showed better physicochemical parameters; four peptides, A1_C, A5_K, A10_C, and A10_M, were found to be stable, nontoxic, and without allergen traits, whereas A10_N was found to be unstable, nontoxic, and nonallergenic. These outstanding parameters are not different from those discussed in a study on milk‐derived peptides, casein, and lactoferrin by Mukhopadhyay [80]. Drako et al. [79] also reported similar findings where a majority of their potential lead compounds had a low possibility of toxicity and allergenicity.

When docked against the Ebola virus VP‐40 receptor using HDOCK and ClusPro, the docking energy scores showed that all five peptides bound with energies ranging from –252.39 to –145.83 kcal/mol and from –887.7 to –538.87 units, respectively, indicating a variation in binding strength among the five peptide–protein complexes. Among the peptides, A5_K exhibited the most favorable binding energy (–252.39 kcal/mol in HDOCK) and a strong ClusPro coefficient (–860.4 units), suggesting high binding affinity and conformational complementarity with the VP‐40 binding site. This implies that A5_K forms a highly stable and energetically favorable complex, likely stabilized by multiple hydrogen bonds, hydrophobic interactions, and electrostatic contacts. Similarly, A10_C demonstrated a strong interaction profile, with a docking energy of –214.91 kcal/mol and the most favorable ClusPro coefficient (–887.7 units). Although its HDOCK score was slightly less negative than that of A5_K, the strong ClusPro coefficient highlights a robust interface and potentially deeper binding pocket engagement. These findings suggest that A10_C, despite minor structural caveats observed during docking, forms a stable and well‐oriented interaction with VP‐40.

The peptides A10_N and A10_M displayed moderate docking affinities, indicating acceptable but less stable peptide–protein interactions compared with A5_K and A10_C. A1_C showed the least favorable binding energy and a weaker ClusPro score, suggesting lower stability and weaker interaction potential at the VP‐40 active site. Overall, both HDOCK and ClusPro results showed consistent ranking trends, identifying A5_K and A10_C as the top‐performing peptides with strong and stable binding orientations toward the VP‐40 protein. In comparison, several in silico studies have also reported similar trends where peptides with more negative docking energies exhibit enhanced stability and strong interface complementarity with Ebola virus proteins. For instance, Li et al. demonstrated in their study that cyclic peptides designed against the Ebola virus glycoprotein (GP) achieved favorable docking stability through persistent hydrogen bonding and hydrophobic contacts, correlating well with inhibitory activity [81]. Likewise, Saxena et al. designed a stapled peptide targeting the VP‐40 dimerization interface and reported stable peptide–protein complexes characterized by extensive electrostatic interactions, reinforcing the potential of peptide‐based inhibitors against Ebola matrix proteins [82]. Additionally, Broni et al. identified small‐molecule VP‐40 inhibitors with docking energies in the range of –200 to –280 kcal/mol, consistent with our observed energy range for high‐affinity peptide binders [83]. These studies, when combined, support our results, which suggest that peptides with the most negative docking scores, like A5_K and A10_C, probably create favorable and stable complexes with VP‐40. The consistency across independent docking platforms and the agreement with published computational studies strengthen the validity of our peptide selection and highlight their potential as promising antiviral candidates against the Ebola virus. Although A10_C achieved the best docking score, structural analysis of the resulting complex in ChimeraX indicated that its binding pocket differed from that of the other peptides. Specifically, A10_C was bound in a smaller pocket, with only a limited region of the peptide engaging the binding site. This suggests that despite its favorable docking score, A10_C may not form a stable or optimal complex for further experimental studies. Additionally, it is important to disregard the ligand RMSD score in this context, as the server treats the peptide as a protein, leading to a high RMSD value.

Upon taking a closer look at the interaction of the complexes, A10_C displayed an extensive interaction network, with hydrogen bonds formed mainly by residues of Pro 97, Asp193, and Lys 212, contributing to polar stabilization of the complex. Hydrophobic contacts reinforced van der Waals complementarity at the binding pocket, indicating that A10_C occupies a deeply buried and energetically favorable interface (Figure 4a). These interactions align with the strong docking energy (–214.91 kcal/mol, ClusPro coefficient weight score –887.7 units), supporting its structural stability and high binding affinity. In contrast, the A10_N exhibited hydrogen bonding with Ala156, Leu218, and Pro286, along with a number of hydrophobic contacts (Figure 4b). Although this pattern indicates a moderately stable interaction, the smaller number of polar contacts may explain the slightly higher (less negative) binding energy (–183.33 kcal/mol, ClusPro –666.3) compared with that of A10_C. The contact maps in our findings confirm that A10_C and A10_N interact with conserved residues located within the VP‐40 oligomerization interface, consistent with previously reported peptide docking studies on Ebola virus matrix proteins [82, 83].

Despite A5_K, A10_N, and A10_C achieving the best docking scores, we conducted MD simulations on all five candidate peptides to better understand their binding interactions with VP‐40. Interestingly, all the complexes maintained structural stability throughout the entire MD simulation trajectory. Especially, A10_M revealed the greatest degree of stability and compactness as evidenced by the consistent RMSD, RMSF, and RoG. This finding is similar to a study by Karthick et al [84] where the potential energy plots obtained from MDS showed that simulated interactions for their lead compounds are stabilized throughout the simulations. Also, in their study, the RMSD results indicate that the movement of lead compounds is small, thereby showing the strength within the binding pockets of VP‐40 [84]. In our study, a comprehensive analysis of the MD trajectories confirmed VP‐40 structural stability with all assessment metrics, including RMSD and RMSF, indicating well‐folded and consistent conformations throughout the simulations.

Peptides present numerous advantages over small molecules that include high biological activity, high specificity, low production cost, and high penetration [37, 85]. However, besides the numerous advantages, peptide stability in serum remains a crucial factor for effective in vivo delivery [36]. Peptides are susceptible to proteolytic degradation in serum, which can limit their half‐life and efficacy [86]. Another important concern is immunogenicity, since exogenous peptides can trigger unwanted immune responses, potentially limiting therapeutic efficacy or safety. Also, they may have rapid clearance rates, which can impact their ability to reach and maintain therapeutic concentrations at the target site [87]. To overcome these barriers, several strategies have been explored, including chemical modifications such as cyclization, stapling, and incorporation of nonnatural amino acids to enhance stability and protease resistance; PEGylation or conjugation with lipids to extend half‐life; and nanoparticle or liposomal delivery systems to improve bioavailability and targeted delivery [88]. Integrating such approaches into future design efforts will be critical for advancing AVPs toward in vivo efficacy and therapeutic application.

The structural stability observed in this study holds important implications for the development of therapeutics against the SEBOV. Our findings establish a foundation for the rational design of antiviral inhibitors targeting the VP‐40 matrix protein of SEBOV. This work advances the field of AVP research by introducing a novel structure‐based design strategy that integrates a de novo peptide library, the AVP database, and multiple complementary computational tools. Through this integrative approach, we identified peptides with enhanced specificity and binding potency toward the SEBOV VP‐40. Moreover, our analyses provide molecular insights into peptide–viral protein interactions, highlighting the key residues and binding modes that contribute to complex stability. These findings not only guide the optimization of peptide‐based antivirals but also open opportunities for developing broad‐spectrum AVPs capable of targeting multiple filoviral strains. Collectively, our study lays the groundwork for the design of effective and selective peptide therapeutics against SEBOV. Clinically, such agents could play a vital role in preventing or treating outbreaks, thereby reducing morbidity and mortality and improving public health outcomes.

Future studies should focus on experimental validation of the promising peptides, elucidating their mechanisms of action, and assessing their efficacy and safety in preclinical and clinical models.

## 5. Conclusions

This study identified five promising AVPs. These properties position them as strong potential candidates for antiviral drug discovery against the SEBOV. However, reliance on computational models and the predicted interactions requires further experimental validation of individual AVP through in vitro and subsequently in vivo studies. In addition, studies exploring strategies to optimize peptide stability and the optimization of peptides against other viruses are suggested.

## Ethics Statement

The authors have nothing to report.

## Disclosure

All the authors have read and approved the final manuscript.

## Conflicts of Interest

The authors declare no conflicts of interest.

## Author Contributions

Boniface Omara: conceptualization, methodology, design, writing the original draft of the manuscript, resources, software, formal analysis, and data curation; Kenedy Kiyimba: writing—review and editing, validation, formal analysis, software, visualization, and resources; Fatoumata G. Fofana: writing—review and editing, validation, formal analysis, software, visualization, and validation; Oudou Diabaté: writing—review and editing, validation, formal analysis, software, visualization, and validation; Walter Odur: Writing—review and editing, software, and visualization; Daudi Jjingo: writing—review and editing, software, and resources; Jacob Stanley Iramiot: writing—review and editing; Peace Draleru: writing—review and editing; Joan Achia: writing—review and editing; Muhammad Shafiq: writing—review and editing, validation, formal analysis, software, visualization, and resources; Zaheer Ul‐Haq: writing—review and editing, validation, formal analysis, software, visualization, and resources; Hedmon Okella and Steven Odongo: writing—review and editing, supervision, conceptualization, data curation.

## Funding

No funding was received for this manuscript.

## Supporting Information

Additional supporting information can be found online in the Supporting Information section.

## Supporting information


**Supporting Information 1** Table S1: A total of 11 Ebola antiviral peptides retrieved from the antiviral peptide database and qualified as experimentally validated.

**Supporting Information 2** Table S2: A total of 170 peptides were designed from the 11 experimentally validated anti‐Ebola peptide sequences using a Support Vector Machine (SVM) classifier at CAMPR_3_ visited at https://www.camp3.bicnirrh.res.in/index.php.

**Supporting Information 3** Table S3: The ligands were modelled using PEPFOLD and I‐TASSER servers. Model_1 for each of the PEPFOLD modelled motifs was recognized as the best model, with their released coarse‐grained optimized potential for efficient structure prediction energy and Apollo melting temperature (tm) scores ranging from −33.33 to −15.19 (sOPEP) and 0.792 to 0.256 (tm), respectively. The I‐TASSER predictions produced five models for A1_C, A10_C, A5_K, three models for A10_M, and one model for A10_N peptides and had c‐scores ranging between −1.47 and −0.01.

**Supporting Information 4** Figure S1: A 3D structure surface‐filled representation of the matrix protein VP‐40 from Sudan Ebolavirus (PDB id: 3tcq). The image was rendered in BIOVIA Discovery Studio 2021 client v21.1.

## Data Availability

The raw data of this study were obtained from the Antiviral Peptide Database (AVPdb) (available at http://crdd.osdd.net/servers/avpd).
