# Supplementary material for: In Silico Identification of Antiviral Peptides as Potential Leads Against Sudan Ebolavirus VP‐40
Source: Biomed Res Int. 2026 Jan 26;2026:2204127. doi: 10.1155/bmri/2204127 (PMC12835197; doi:10.1155/bmri/2204127)
Supplement: Supplementary file 1 — Supporting Information 1 Table S1: A total of 11 Ebola antiviral peptides retrieved from the antiviral peptide database and qualified as experimentally validated. [file BMRI-2026-2204127-s001.docx]

**Supplementary Table S1: Retrieved EBOV AVPs from the AVPdb**

| **AVP Id** | **Name of Peptide** | **Length** | **Source** | **Reference** |
| --- | --- | --- | --- | --- |
| AVP0210 | E30 pep-wt | 23 | EboV VP30 protein | [1] |
| AVP1117 | EBOV C-Peptide | 38 | EboV C protein/HIV Tat | [2] |
| AVP 1588 | Fc-tagged delta peptide | 48 | EboV delta peptide | [3] |
| AVP 1589 | Fc-tagged delta peptide | 28 | EboV delta peptide | [3] |
| AVP 1560 | Fc-tagged delta peptide | 33 | EboV delta peptide | [3] |
| AVP 1561 | Fc-tagged delta peptide | 39 | EboV delta peptide | [3] |
| AVP 1562 | Fc-tagged delta peptide | 36 | EboV delta peptide | [3] |
| AVP 1563 | Fc-tagged delta peptide | 21 | EboV delta peptide | [3] |
| AVP 1564 | Fc-tagged delta peptide | 42 | EboV delta peptide | [3] |
| AVP 1565 | Fc-tagged delta peptide | 31 | EboV delta peptide | [3] |
| AVP 1567 | Fc-tagged delta peptide | 17 | EboV delta peptide | [3] |

**AVP ID:** Antiviral Peptide Identity; **AVPdb**: Antiviral Peptide database; **EBOV:** *Ebolavirus,* **AVPs:** *antiviral peptides*

**References**

[1] B. Hartlieb, J. Modrof, E. Mühlberger, H.-D. Klenk, and S. Becker, “Oligomerization of Ebola Virus VP30 Is Essential for Viral Transcription and Can Be Inhibited by a Synthetic Peptide *,” *J. Biol. Chem.*, vol. 278, no. 43, pp. 41830–41836, Oct. 2003, doi: 10.1074/jbc.M307036200.

[2] C. K. Miller EH, Harrison JS, Radoshitzky SR, Higgins CD, Chi X, Dong L, Kuhn JH, Bavari S, Lai JR, “Inhibition of Ebola virus entry by a C-peptide targeted to endosomes.,” *J Biol Chem*, pp. 286(18):15854–61, 2011.

[3] K. J. Radoshitzky SR, Warfield KL, Chi X, Dong L, Kota K, Bradfute SB, Gearhart JD, Retterer C, Kranzusch PJ, Misasi JN, Hogenbirk MA, Wahl-Jensen V, Volchkov VE, Cunningham JM, Jahrling PB, Aman MJ, Bavari S, Farzan M, “Ebolavirus delta-peptide immunoadhesins inhibit marburgvirus and ebolavirus cell entry.,” *J Virol. 2011*, 2011.
