# Supplementary material for: In Silico Identification of Antiviral Peptides as Potential Leads Against Sudan Ebolavirus VP‐40
Source: Biomed Res Int. 2026 Jan 26;2026:2204127. doi: 10.1155/bmri/2204127 (PMC12835197; doi:10.1155/bmri/2204127)
Supplement: Supplementary file 2 — Supporting Information 2 Table S2: A total of 170 peptides were designed from the 11 experimentally validated anti‐Ebola peptide sequences using a Support Vector Machine (SVM) classifier at CAMPR3 visited at https://www.camp3.bicnirrh.res.in/index.php. [file BMRI-2026-2204127-s002.docx]

**Supplementary Table S2: Generated motif sequences with Support Vector Machine (SVM) classifier**

| **S/No** | **Peptide ID** | **Position** | **Sequence** |
| --- | --- | --- | --- |
| 1 | A1_A | 1-18 | QLESLTDRELLLLIARKT |
| 2 | A1_B | 2-19 | LESLTDRELLLLIARKTC |
| 3 | A1_C | 3-20 | ESLTDRELLLLIARKTCG |
| 4 | A1_D | 4-21 | SLTDRELLLLIARKTCGS |
| 5 | A1_E | 5-22 | LTDRELLLLIARKTCGSV |
| 6 | A1_F | 6-23 | TDRELLLLIARKTCGSVE |
| 7 | A2_A | 1-18 | YGRKKRRQRRRGSGIEPH |
| 8 | A2_B | 2-19 | GRKKRRQRRRGSGIEPHD |
| 9 | A2_C | 3-20 | RKKRRQRRRGSGIEPHDW |
| 10 | A2_D | 4-21 | KKRRQRRRGSGIEPHDWT |
| 11 | A2_E | 5-22 | KRRQRRRGSGIEPHDWTK |
| 12 | A2_F | 6-23 | RRQRRRGSGIEPHDWTKN |
| 13 | A2_G | 7-24 | RQRRRGSGIEPHDWTKNI |
| 14 | A2_H | 8-25 | QRRRGSGIEPHDWTKNIT |
| 15 | A2_I | 9-26 | RRRGSGIEPHDWTKNITD |
| 16 | A2_J | 10-27 | RRGSGIEPHDWTKNITDK |
| 17 | A2_K | 11-28 | RGSGIEPHDWTKNITDKI |
| 18 | A2_L | 12-29 | GSGIEPHDWTKNITDKID |
| 19 | A2_M | 13-30 | SGIEPHDWTKNITDKIDQ |
| 20 | A2_N | 14-31 | GIEPHDWTKNITDKIDQI |
| 21 | A2_O | 15-32 | IEPHDWTKNITDKIDQII |
| 22 | A2_P | 16-33 | EPHDWTKNITDKIDQIIH |
| 23 | A2_Q | 17-34 | PHDWTKNITDKIDQIIHD |
| 24 | A2_R | 18-35 | HDWTKNITDKIDQIIHDF |
| 25 | A2_S | 19-36 | DWTKNITDKIDQIIHDFV |
| 26 | A2_T | 20-37 | WTKNITDKIDQIIHDFVD |
| 27 | A2_W | 21-38 | TKNITDKIDQIIHDFVDK |
| 28 | A3_A | 1-18 | ELQREESPTGPPGSIRTW |
| 29 | A3_B | 2-19 | LQREESPTGPPGSIRTWF |
| 30 | A3_C | 3-20 | QREESPTGPPGSIRTWFQ |
| 31 | A3_D | 4-21 | REESPTGPPGSIRTWFQR |
| 32 | A3_E | 5-22 | EESPTGPPGSIRTWFQRI |
| 33 | A3_F | 6-23 | ESPTGPPGSIRTWFQRIP |
| 34 | A3_G | 7-24 | SPTGPPGSIRTWFQRIPL |
| 35 | A3_H | 8-25 | PTGPPGSIRTWFQRIPLG |
| 36 | A3_I | 9-26 | TGPPGSIRTWFQRIPLGW |
| 37 | A3_J | 10-27 | GPPGSIRTWFQRIPLGWF |
| 38 | A3_K | 11-28 | PPGSIRTWFQRIPLGWFH |
| 39 | A3_L | 12-29 | PGSIRTWFQRIPLGWFHC |
| 40 | A3_M | 13-30 | GSIRTWFQRIPLGWFHCT |
| 41 | A3_N | 14-31 | SIRTWFQRIPLGWFHCTY |
| 42 | A3_O | 15-32 | IRTWFQRIPLGWFHCTYQ |
| 43 | A3_P | 16-33 | RTWFQRIPLGWFHCTYQK |
| 44 | A3_Q | 17-34 | TWFQRIPLGWFHCTYQKG |
| 45 | A3_R | 18-35 | WFQRIPLGWFHCTYQKGK |
| 46 | A3_S | 19-36 | FQRIPLGWFHCTYQKGKQ |
| 47 | A3_T | 20-37 | QRIPLGWFHCTYQKGKQH |
| 48 | A3_U | 21-38 | RIPLGWFHCTYQKGKQHC |
| 49 | A3_V | 22-39 | IPLGWFHCTYQKGKQHCR |
| 50 | A3_W | 23-40 | PLGWFHCTYQKGKQHCRL |
| 51 | A3_X | 24-41 | LGWFHCTYQKGKQHCRLR |
| 52 | A3_Y | 25-42 | GWFHCTYQKGKQHCRLRI |
| 53 | A3_Z | 26-43 | WFHCTYQKGKQHCRLRIR |
| 54 | A3_ZA | 27-44 | FHCTYQKGKQHCRLRIRQ |
| 55 | A3_ZB | 28-45 | HCTYQKGKQHCRLRIRQK |
| 56 | A3_ZC | 29-46 | CTYQKGKQHCRLRIRQKV |
| 57 | A3_ZD | 30-47 | TYQKGKQHCRLRIRQKVE |
| 58 | A3_ZE | 31-48 | YQKGKQHCRLRIRQKVEE |
| 59 | A4_A | 1-18 | ELQREESPTGPPGSIRTW |
| 60 | A4_B | 2-19 | LQREESPTGPPGSIRTWF |
| 61 | A4_C | 3-20 | QREESPTGPPGSIRTWFQ |
| 62 | A4_D | 4-21 | REESPTGPPGSIRTWFQR |
| 63 | A4_E | 5-22 | EESPTGPPGSIRTWFQRI |
| 64 | A4_F | 6-23 | ESPTGPPGSIRTWFQRIP |
| 65 | A4_G | 7-24 | SPTGPPGSIRTWFQRIPL |
| 66 | A4_H | 8-25 | PTGPPGSIRTWFQRIPLG |
| 67 | A4_I | 9-26 | TGPPGSIRTWFQRIPLGW |
| 68 | A4_J | 10-27 | GPPGSIRTWFQRIPLGWF |
| 69 | A4_K | 11-28 | PPGSIRTWFQRIPLGWFH |
| 70 | A5_A | 6-23 | ESPTGPPGSIRTWFQRIP |
| 71 | A5_B | 10-27 | GPPGSIRTWFQRIPLGWF |
| 72 | A5_C | 13-20 | GSIRTWFQRIPLGWFHCT |
| 73 | A5_D | 5-22 | EESPTGPPGSIRTWFQRI |
| 74 | A5_E | 12-19 | PGSIRTWFQRIPLGWFHC |
| 75 | A5_F | 4-21 | REESPTGPPGSIRTWFQR |
| 76 | A5_G | 11-28 | PPGSIRTWFQRIPLGWFH |
| 77 | A5_H | 9-26 | TGPPGSIRTWFQRIPLGW |
| 78 | A5_I | 16-33 | RTWFQRIPLGWFHCTYQK |
| 79 | A5_J | 3-20 | QREESPTGPPGSIRTWFQ |
| 80 | A5_K | 14-31 | SIRTWFQRIPLGWFHCTY |
| 81 | A5_L | 8-25 | PTGPPGSIRTWFQRIPLG |
| 82 | A5_M | 15-32 | IRTWFQRIPLGWFHCTYQ |
| 83 | A5_N | 7-24 | SPTGPPGSIRTWFQRIPL |
| 84 | A5_O | 2-19 | LQREESPTGPPGSIRTWF |
| 85 | A5_P | 1-18 | ELQREESPTGPPGSIRTW |
| 86 | A6_A | 6-23 | ESPTGPPGSIRTWFQRIP |
| 87 | A6_B | 10-27 | GPPGSIRTWFQRIPLGWF |
| 88 | A6_C | 13-30 | GSIRTWFQRIPLGWFHCT |
| 89 | A6_D | 5-22 | EESPTGPPGSIRTWFQRI |
| 90 | A6_E | 21-38 | RIPLGWFHCTYQKGKQHC |
| 91 | A6_F | 12-29 | PGSIRTWFQRIPLGWFHC |
| 92 | A6_G | 4-21 | REESPTGPPGSIRTWFQR |
| 93 | A6_H | 11-28 | PPGSIRTWFQRIPLGWFH |
| 94 | A6_I | 19-36 | FQRIPLGWFHCTYQKGKQ |
| 95 | A6_J | 18-35 | WFQRIPLGWFHCTYQKGK |
| 96 | A6_K | 9-26 | TGPPGSIRTWFQRIPLGW |
| 97 | A6_L | 22-39 | IPLGWFHCTYQKGKQHCR |
| 98 | A6_M | 16-33 | RTWFQRIPLGWFHCTYQK |
| 99 | A6_N | 20-37 | QRIPLGWFHCTYQKGKQH |
| 100 | A6_O | 3-20 | QREESPTGPPGSIRTWFQ |
| 101 | A6_P | 14-31 | SIRTWFQRIPLGWFHCTY |
| 102 | A6_Q | 8-25 | PTGPPGSIRTWFQRIPLG |
| 103 | A6_R | 15-32 | IRTWFQRIPLGWFHCTYQ |
| 104 | A6_S | 17-34 | TWFQRIPLGWFHCTYQKG |
| 105 | A6_T | 7-24 | SPTGPPGSIRTWFQRIPL |
| 106 | A6_U | 2-19 | LQREESPTGPPGSIRTWF |
| 107 | A6_V | 1-18 | ELQREESPTGPPGSIRTW |
| 108 | A7_A | 1-18 | GSIRTWFQRIPLGWFHCT |
| 109 | A7_B | 2-19 | SIRTWFQRIPLGWFHCTY |
| 110 | A7_C | 3-20 | IRTWFQRIPLGWFHCTYQ |
| 111 | A7_D | 4-21 | RTWFQRIPLGWFHCTYQK |
| 112 | A7_E | 5-22 | TWFQRIPLGWFHCTYQKG |
| 113 | A7_F | 6-23 | WFQRIPLGWFHCTYQKGK |
| 114 | A7_G | 7-24 | FQRIPLGWFHCTYQKGKQ |
| 115 | A7_H | 8-25 | QRIPLGWFHCTYQKGKQH |
| 116 | A7_I | 9-26 | RIPLGWFHCTYQKGKQHC |
| 117 | A7_J | 10-27 | IPLGWFHCTYQKGKQHCR |
| 118 | A7_K | 11-28 | PLGWFHCTYQKGKQHCRL |
| 119 | A7_L | 12-29 | LGWFHCTYQKGKQHCRLR |
| 120 | A7_M | 13-30 | GWFHCTYQKGKQHCRLRI |
| 121 | A7_N | 14-31 | WFHCTYQKGKQHCRLRIR |
| 122 | A7_O | 15-32 | FHCTYQKGKQHCRLRIRQ |
| 123 | A7_P | 16-33 | HCTYQKGKQHCRLRIRQK |
| 124 | A7_Q | 17-34 | CTYQKGKQHCRLRIRQKV |
| 125 | A7_R | 18-35 | TYQKGKQHCRLRIRQKVE |
| 126 | A7_S | 19-36 | YQKGKQHCRLRIRQKVEE |
| 127 | A8_A | 1-18 | HCTYQKGKQHCRLRIRQK |
| 128 | A8_B | 2-19 | CTYQKGKQHCRLRIRQKV |
| 129 | A8_C | 3-20 | TYQKGKQHCRLRIRQKVE |
| 130 | A8_D | 4-21 | YQKGKQHCRLRIRQKVEE |
| 131 | A9_A | 1-18 | SPTGPPGSIRTWFQRIPL |
| 132 | A9_B | 2-19 | PTGPPGSIRTWFQRIPLG |
| 133 | A9_C | 3-20 | TGPPGSIRTWFQRIPLGW |
| 134 | A9_D | 4-21 | GPPGSIRTWFQRIPLGWF |
| 135 | A9_E | 5-22 | PPGSIRTWFQRIPLGWFH |
| 136 | A9_F | 6-23 | PGSIRTWFQRIPLGWFHC |
| 137 | A9_G | 7-24 | GSIRTWFQRIPLGWFHCT |
| 138 | A9_H | 8-25 | SIRTWFQRIPLGWFHCTY |
| 139 | A9_I | 9-26 | IRTWFQRIPLGWFHCTYQ |
| 140 | A9_J | 10-27 | RTWFQRIPLGWFHCTYQK |
| 141 | A9_K | 11-28 | TWFQRIPLGWFHCTYQKG |
| 142 | A9_L | 12-29 | WFQRIPLGWFHCTYQKGK |
| 143 | A9_M | 13-30 | FQRIPLGWFHCTYQKGKQ |
| 144 | A9_N | 14-31 | QRIPLGWFHCTYQKGKQH |
| 145 | A9_O | 15-32 | RIPLGWFHCTYQKGKQHC |
| 146 | A9_P | 16-33 | IPLGWFHCTYQKGKQHCR |
| 147 | A9_Q | 17-34 | PLGWFHCTYQKGKQHCRL |
| 148 | A9_R | 18-35 | LGWFHCTYQKGKQHCRLR |
| 149 | A9_S | 19-36 | GWFHCTYQKGKQHCRLRI |
| 150 | A9_T | 20-37 | WFHCTYQKGKQHCRLRIR |
| 151 | A9_U | 21-38 | FHCTYQKGKQHCRLRIRQ |
| 152 | A9_V | 22-39 | HCTYQKGKQHCRLRIRQK |
| 153 | A9_W | 23-40 | CTYQKGKQHCRLRIRQKV |
| 154 | A9_X | 24-41 | TYQKGKQHCRLRIRQKVE |
| 155 | A9_Y | 25-42 | YQKGKQHCRLRIRQKVEE |
| 156 | A10_A | 1-18 | WFQRIPLGWFHCTYQKGK |
| 157 | A10_B | 2-19 | FQRIPLGWFHCTYQKGKQ |
| 158 | A10_C | 3-20 | QRIPLGWFHCTYQKGKQH |
| 159 | A10_D | 4-21 | RIPLGWFHCTYQKGKQHC |
| 160 | A10_E | 5-22 | IPLGWFHCTYQKGKQHCR |
| 161 | A10_F | 6-23 | PLGWFHCTYQKGKQHCRL |
| 162 | A10_G | 7-24 | LGWFHCTYQKGKQHCRLR |
| 163 | A10_H | 8-25 | GWFHCTYQKGKQHCRLRI |
| 164 | A10_I | 9-26 | WFHCTYQKGKQHCRLRIR |
| 165 | A10_J | 10-27 | FHCTYQKGKQHCRLRIRQ |
| 166 | A10_K | 11-28 | HCTYQKGKQHCRLRIRQK |
| 167 | A10_L | 12-29 | CTYQKGKQHCRLRIRQKV |
| 168 | A10_M | 13-30 | TYQKGKQHCRLRIRQKVE |
| 169 | A10_N | 14-31 | YQKGKQHCRLRIRQKVEE |
| 170 | A11_A | 1-18 | ELQREESPTGPPGSIRT |

**Peptide Id;** Peptide Identity
