# Supplementary material for: In Silico Identification of Antiviral Peptides as Potential Leads Against Sudan Ebolavirus VP‐40
Source: Biomed Res Int. 2026 Jan 26;2026:2204127. doi: 10.1155/bmri/2204127 (PMC12835197; doi:10.1155/bmri/2204127)
Supplement: Supplementary file 3 — Supporting Information 3 Table S3: The ligands were modelled using PEPFOLD and I‐TASSER servers. Model_1 for each of the PEPFOLD modelled motifs was recognized as the best model, with their released coarse‐grained optimized potential for efficient structure prediction energy and Apollo melting temperature (tm) scores ranging from −33.33 to −15.19 (sOPEP) and 0.792 to 0.256 (tm), respectively. The I‐TASSER predictions produced five models for A1_C, A10_C, A5_K, three models for A10_M, and one model for A10_N peptides and had c‐scores ranging between −1.47 and −0.01. [file BMRI-2026-2204127-s004.docx]

**Supplementary Table S3: Top 5 output peptide structure prediction models from I-TASSER, PEP-FOLD, and their model evaluation**

| **I-TASSER output Model c-score** | | **1** | **2** | **3** | **4** | **5** |
| --- | --- | --- | --- | --- | --- | --- |
| **A1_C** |  | -0.45 | -5.00 | -3.91 | -3.76 | -5.00 |
| **A5_K** |  | -1.07 | -1.15 | -2.92 | -5.00 | -5.00 |
| **A10_C** |  | -1.47 | -2.41 | -3.36 | -2.39 | -1.98 |
| **A10_M** |  | -0.01 | -5.00 | -5.00 | - | - |
| **A10_N** |  | -0.05 | - | - | - | - |
| **PEP-FOLD output model scores 1 2 3 4 5** | | | | | | |
| **A1_C** | sOPEP | -33.3250 | -33.0626 | -31.8433 | -30.7010 | -30.3123 |
|  | tm | 0.792 | 0.743 | 0.739 | 0.656 | 0.0.603 |
| **A5_K** | sOPEP | -31.0343 | -30.1466 | -30.0348 | -29.9716 | -29.9113 |
|  | t_m_ | 0.461 | 0.468 | 0.424 | 0.405 | 0.416 |
| **A10_C** | sOPEP | -15.1870 | 14.6220 | -14.2380 | -14.0787 | -13.3604 |
|  | t_m_ | 0.256 | 0.289 | 0.303 | 0.277 | 0.297 |
| **A10_M** | sOPEP | -26.4148 | -23.5437 | -22.4432 | -21.3851 | -21.3370 |
|  | t_m_ | 0.422 | 0.358 | 0.429 | 0.401 | 0.401 |
| **A10_N** | sOPEP | -29.3112 | -25.9116 | -25.3531 | -25.4309 | -25.3874 |
|  | t_m_ | 0.447 | 0.439 | 0.420 | 0.428 | 0.443 |

**t_m_**, Apollo melting temperature; **sOPEP**, Optimized Potential for Efficient Energy Prediction
