# Supplementary figures and images for: In Silico Identification of Antiviral Peptides as Potential Leads Against Sudan Ebolavirus VP‐40
Source: Biomed Res Int. 2026 Jan 26;2026:2204127. doi: 10.1155/bmri/2204127 (PMC12835197; doi:10.1155/bmri/2204127)

## Slide 1
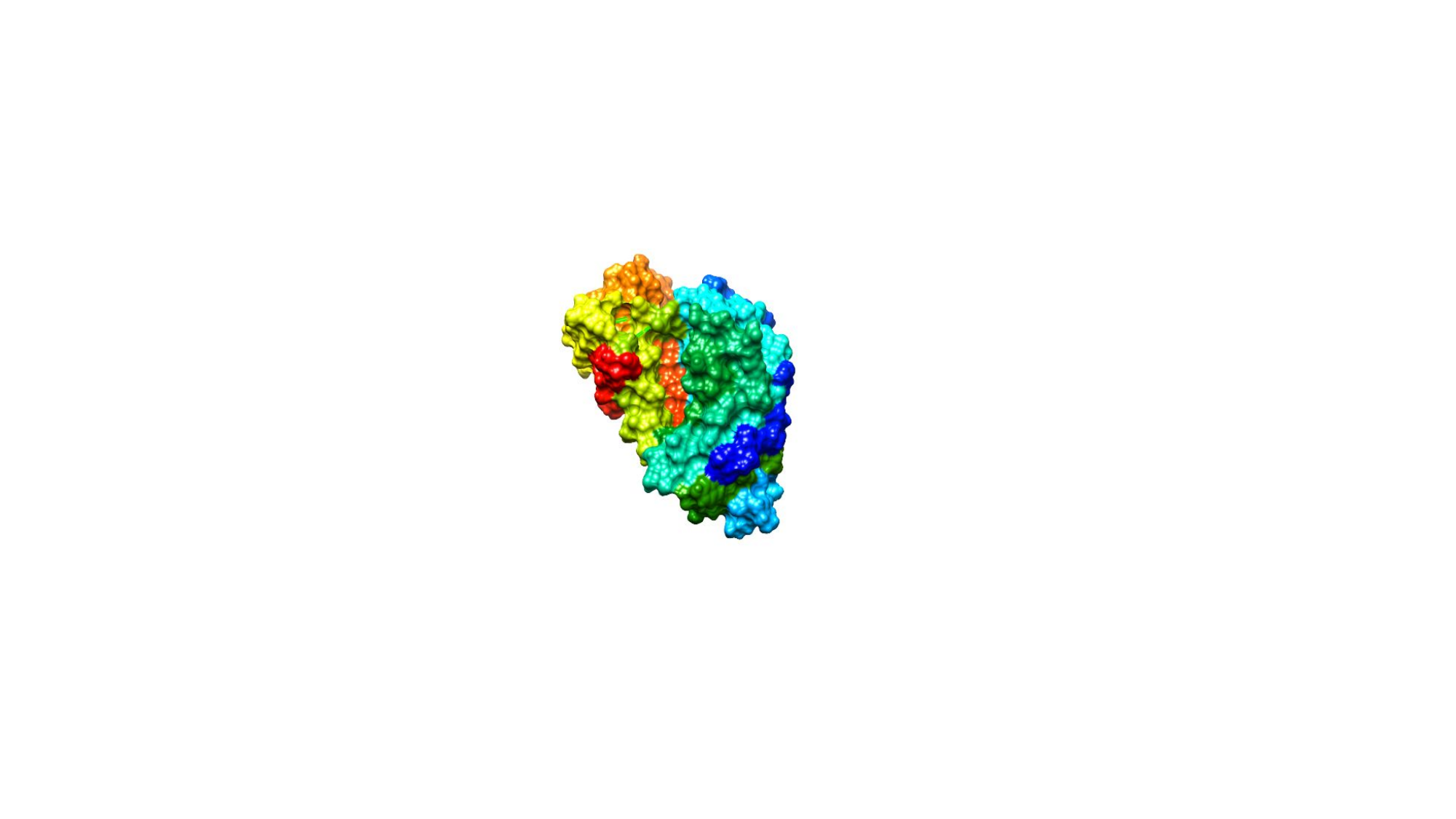

Supplement: Supplementary file 4 — Supporting Information 4 Figure S1: A 3D structure surface‐filled representation of the matrix protein VP‐40 from Sudan Ebolavirus (PDB id: 3tcq). The image was rendered in BIOVIA Discovery Studio 2021 client v21.1. [file BMRI-2026-2204127-s003.pptx]
